# Ways to increase equity, diversity and inclusion

**DOI:** 10.7554/eLife.60438

**Published:** 2020-07-07

**Authors:** Devang Mehta, Yaw Bediako, Charlotte M de Winde, Hedyeh Ebrahimi, Florencia Fernández-Chiappe, Vinodh Ilangovan, Carolina Paz Quezada, Julia L Riley, Shyam M Saladi, Andy Tay, Tracey Weissgerber

**Affiliations:** 1 Department of Biological Sciences, University of AlbertaEdmontonCanada; 2 West African Centre for Cell Biology of Infectious Pathogens, University of GhanaAccraGhana; 3 MRC Laboratory for Molecular Cell Biology, University College LondonLondonUnited Kingdom; 4 Non-Communicable Diseases Research Center, Endocrinology and Metabolism Population Sciences InstituteTehranIslamic Republic of Iran; 5 Instituto de Investigación en Biomedicina de Buenos Aires – CONICET – Partner Institute of the Max Planck SocietyBuenos AiresArgentina; 6 Aarhus UniversityAarhusDenmark; 7 Centro Integrativo de Biología y Química Aplicada, Universidad Bernardo O'HigginsSantiagoChile; 8 Department of Botany and Zoology, Stellenbosch UniversityStellenboschSouth Africa; 9 Department of Biology, Dalhousie UniversityHalifaxCanada; 10 Biochemistry and Molecular Biophysics Option, California Institute of TechnologyPasadenaUnited States; 11 Department of Biomedical Engineering, National University of SingaporeSingaporeSingapore; 12 QUEST – Quality | Ethics | Open Science | Translation, Charité – Universitätsmedizin Berlin, Berlin Institutes of HealthBerlinGermany

**Keywords:** early-career researchers, equity, diversity and inclusion, scientific publishing, peer review

## Abstract

The eLife Early-Career Advisory Group (ECAG), an international group of early-career researchers committed to improving research culture, calls for radical changes at eLife and other journals to address racism in the scientific community and to make science more diverse and inclusive.

In recent weeks, following the growth of the Black Lives Matter movement, several scientific organizations, including eLife, have spoken out against systemic racism in science (eLife, 2020a). However, this acknowledgement comes too late for many, given that the scientific community was largely silent over the past year following a number of similarly shocking events in Iran (when hundreds of protestors, including students, were killed; McKenzie, 2019), in Chile (when thousands were jailed; Boddenberg and McGown, 2020), and in India (when police invaded university libraries to assault students; Bhandari, 2019). Our silence shames us. We believe the freedom to think and to share ideas without the threat of discrimination and violence is essential to the development of science. It is the duty of scientific organizations to defend these freedoms when they are threatened anywhere on the planet.

It is not lost on us that, as life scientists, we have a special responsibility to be anti-racist due to the role our community has played in the past by providing quasi-scientific cover (Saini, 2019) for racist ideologies, from endorsing eugenics (Wilson, 2014) to honoring James Watson, a scientist with a history of expressing racist and sexist views (Belluz, 2019). We are living through a clarifying moment that has clearly galvanized the scientific world in recognition and support for combatting the systemic racism in our fields. We promise to use this new energy and focus to advance innovations at eLife that will lead to a more equitable research culture. We hope that other journals and organizations will take similar actions.

Our role at eLife is to advocate for the inclusion of early-career scientists in the research communication landscape. In many ways, due to the diversity gap between early-career researchers and senior scientists, this mission also involves advocating for equity, diversity and inclusion. Indeed, we strive to ensure representation in terms of gender, ethnicity, and geography within the ECAG (which currently has 11 members) and the much larger eLife Community Ambassadors program, which has hundreds of members (eLife, 2019a), and we will continue to do so. Our mission includes ensuring fair and just outcomes in peer review, and increasing the diversity of the journal's editorial boards and reviewer pool. We have had mixed success to date and we know that we can do better.

It is well known that peer review suffers from a number of biases. eLife's own data have shown the detrimental effect of reviewer homophily (the tendency of reviewers to be more positive about papers by authors of the same gender or from the same country) in editorial decisions for women and minority scientists (Murray et al., 2019). And a different eLife study has found evidence of bias on the grounds of career stage and geography (eLife, 2019b). The journal has begun taking action to address these issues, such as increasing the representation of female scientists on the Board of Reviewing Editors (eLife, 2020b), and there are efforts to increase the use of early-career researchers as editors and reviewers (eLife, 2019c) that date back to 2016 (in response to advice from ECAG; eLife, 2016).

ECAG and eLife also support early-career researchers from low- and middle-income countries through the annual Ben Barres Spotlight Award, and by ensuring that eLife Early-Career Travel Grants are awarded to a diverse group of authors. More recently, eLife has publicly committed to addressing racism in science (Eisen, 2020), including specific measures to address the lack of Black scientists at all levels of the organization. However, there is still a long way to go. So today, we wish to publicly share the specific policies that we ask eLife to adopt and that will lead to lasting change for minority scientists in research communication. We think that being transparent about our objectives will focus our efforts and allow the scientific community to hold us to account. We will push for the adoption of these ideas and policies at eLife and at other organizations involved in shaping the future of science. We urge other journals and scientific organizations to adopt similar measures.

- Include underrepresented minority scientists in the eLife editorial leadership team, which is currently all white. We ask eLife to address the relative lack of diversity at all levels at the journal.
- Report publicly, ideally on a quarterly basis, on progress towards meeting the commitments made in Eisen, 2020, the recommendations in this article, and any future commitments and recommendations. These reports should also contain current and historic demographic data on the composition of the Board of Reviewing Editors and Senior Editors, and eLife's existing targets for representation on the Board of Reviewing Editors by gender, geography and career stage.
- Recruit new editors through open calls aimed at meeting the diversity targets set above.
- Take measures to ensure that editors avoid homophily by, for example, setting a target for the percentage of papers to be evaluated by at least one female reviewer and/or at least one early-career reviewer by a certain date.
- Increase the number of reviewers in the existing database of early-career reviewers, ensure that this database is diverse, and both encourage and make it easier for editors to use this database to recruit reviewers. ECAG and the eLife Community Ambassadors are ready to assist with populating this database.
- Implement systems that report to all editors their record of recruiting diverse reviewer panels. Evaluate the performance of editors based on their use of diverse reviewer panels.
- Offer either mandatory implicit bias training, or racism and sexism awareness-raising workshops for ECAG, Senior Editors, leadership and staff.
- Take clear and vocal editorial positions on sociopolitical issues that affect scientists, especially in situations where eLife can take meaningful action.
- Prioritize the technological and infrastructure innovations required to achieve these objectives.

We also ask eLife to address the relative lack of diversity on the executive staff in the Cambridge office, and to review existing ties to external contractors and ensure that such relationships are consistent with eLife's values.

Minority scientists, including those on the ECAG, sometimes hear that policies to address discriminatory outcomes in science may lead to a lowering of standards, but these assertions are never accompanied by evidence, and may even run counter to it (Nature, 2018). Further, we believe that eLife's committed editors will embrace opportunities that advance the organization’s mission to promote responsible behavior. Too often, leaders in science make arguments against reform that are based on the flawed assumption that the status quo is at all tolerable to the communities that they represent. We urge the leadership at eLife and in other scientific organizations to recognize that current systems in science, from publishing to career advancement, do not work for minority scientists. We ask leaders in science to not underestimate the deep-seated commitment to work for progress that exists among the global community of early-career researchers.

The eLife Early-Career Advisory Group stands ready to continue to dedicate our time to assist eLife's staff and leadership in achieving these objectives and living up to our shared values. We remain assured that the eLife Community Ambassadors and the journal's global team of more than 600 Senior and Reviewing Editors will work together for just outcomes in publishing and contribute to improving research culture. eLife is undergoing a period of transformation and we look forward to helping the organization establish an example for publishing and promoting responsible, fair, and equitable science that inspires others.
